# Systemic Pseudohypoaldosteronism Type I: A Case Report and Review of the Literature

**DOI:** 10.1155/2017/7939854

**Published:** 2017-04-18

**Authors:** Nasifa Nur, Cameron Lang, Juanita K. Hodax, Jose Bernardo Quintos

**Affiliations:** ^1^Division of Pediatric Endocrinology, Rhode Island Hospital and The Warren Alpert Medical School of Brown University, Providence, RI, USA; ^2^Department of Pediatrics, University of North Carolina School of Medicine, Chapel Hill, NC, USA

## Abstract

Systemic pseudohypoaldosteronism (PHA) type I is a rare genetic disorder resulting from mutations in the subunits of the epithelial sodium channel that manifests as severe salt wasting, hyperkalemia, and metabolic acidosis in infancy. In this article we report a patient with systemic PHA type I presenting with severe dehydration due to salt wasting at 6 days of life. She was found to have a known mutation in the SCNN1A gene and subsequently required treatment with sodium supplementation. We also review the clinical presentation, differential diagnosis, and treatment of systemic PHA type I and summarize data from 27 cases with follow-up data.

## 1. Introduction

Pseudohypoaldosteronism (PHA) type I is a rare salt wasting syndrome caused by aldosterone resistance with two distinct forms: an autosomal dominant type, caused by a mutation of the mineralocorticoid receptor with sodium wasting in the kidney, and an autosomal recessive form, resulting from mutations in one of the 3 subunits (alpha, beta, or gamma) of the epithelial Na^+^ channel (ENaC) [1, 2]. The ENaC channel allows the flow of sodium from the lumen into the epithelial cell and modulates the amount of sodium in the extracellular fluid [2]. The autosomal dominant form, also known as renal PHA type I, is confined to the kidney with less severe disease and no systemic involvement. In the autosomal recessive form, also known as systemic PHA type I, patients have multisystem disease with sodium wasting in the kidney, lungs, colon, sweat, and salivary glands. As a result of decreased sodium-dependent liquid absorption in the lungs, children often develop pulmonary symptoms including congestion, wheezing, and recurrent pulmonary infections. Renal PHA type I is generally milder and tends to remit with age, whereas systemic PHA type I persists and requires lifelong treatment [2–4]. Cheek and Perry first described PHA in 1958 [5]. Since then, several patients have been reported and our understanding of the disease has expanded dramatically. Here we report a patient with systemic PHA type I and review the literature on this disease including the clinical presentation, differential diagnosis, genetic diagnosis, treatment, and follow-up data.

## 2. Case Report

A 6-day-old girl presented with vomiting, lethargy, and severe dehydration. Physical examination showed an ill appearing infant with pale skin, poor perfusion, and sunken eyes. She was born full-term with birth weight 3.0 kg to consanguineous parents of Pakistani origin, with pregnancy complicated only by gestational diabetes requiring insulin. She had normal genitalia without clitoromegaly, labial fusion, or hyperpigmentation and no palpable abdominal mass. Laboratory evaluation showed hyponatremia Na^+^ 124 mEq/L, hyperkalemia K^+^ 10 mEq/L, metabolic acidosis HCO_3_^−^ 10 mEq/L, urine Na^+^ 150 mEq/L, elevated serum aldosterone 8.97 nmol/L (normal range 0–6 nmol/L for term infants), plasma renin activity 13.39 ng/mL/hr (normal range 2–35 ng/mL/hr for infants 1–7 days, though some labs use reference range 1.4–7.8 ng/mL/hr for this age), normal serum cortisol 634.8 nmol/L (normal >500 nmol/L during acute stress), and normal 17-hydroxyprogesterone 0.7 nmol/L (normal range 0.2–2.3 nmol/L for term infants). A sweat test showed elevated sweat Cl^−^ 34 mEq/L (normal range 0–29 mEq/L for infants 0–4 months). She had a normal renal ultrasound.

She required two normal saline boluses, oxygen, and several doses of Kayexalate and NaHCO_3_ as well as an insulin drip and glucose to correct electrolyte abnormalities. Sodium and potassium normalized within 2 days. She subsequently developed right lower lobe pneumonia requiring antibiotics and intubation, as well as Klebsiella and Enterococcus sepsis from her femoral line. She was discharged from the hospital after 4 weeks but then developed ventricular tachycardia requiring cardioversion due to hyperkalemia with K^+^ >9 mEq/L. Potassium levels normalized after increasing Kayexalate. She was then maintained on sodium supplementation of 49 mEq/kg/day as well as Kayexalate 3 grams every 8 hours.

Full sequencing of the SCNN1A gene that encodes the alpha subunit of ENaC was performed at Johns Hopkins University. Reported test methods were DNA extraction from whole blood, amplification of the coding sequence of SCNN1G by polymerase chain reaction in 14 segments, cycle sequencing of each segment in the forward and reverse directions, and analysis of each sequence by capillary electrophoresis. This showed a known mutation Nt1685C->T causing replacement of leucine for serine at position 562 in exon 13, which forms the 2nd transmembrane subunit of the alpha chain of ENaC. This mutation was previously reported in a patient with systemic PHA type I [11] and confirmed the diagnosis in our patient. The testing was unable to determine if the mutation was present in two copies (homozygous) or one copy with a deletion of the 2nd allele (hemizygous). Sequencing of parental genes was not done.

The patient began developing the characteristic pulmonary symptoms at age of 2 years with recurrent pneumonia related to chronic bronchorrhea requiring intermittent treatment with Atrovent (Ipratropium). She had 7 episodes of pneumonia between ages of 2 and 3 years. These symptoms improved with age and frequency of infections decreased. Repeat sweat test at age of 3 years showed elevated sweat Cl^−^ 133 mEq/L (normal range 0–39 mEq/L for patients >4 months). At her last follow-up at 5 and 5/12 years of age, she was receiving Kayexalate, NaCl, and NaHCO_3_ for a total dose of 23 mEq/kg/day of sodium. She has not had any recurrent salt wasting crises. Her height was 103.8 cm (−1.5 SDS), weight 13.4 kg (−3.1 SDS), and BMI 12.4 kg/m^2^ (−3.2 SDS). Labs at that time showed normal electrolytes with Na^+^ 135 mEq/L and K^+^ 4.4 mEq/L.

The patient had 2 younger siblings born to the same mother and father. The first sibling was clinically unaffected, and genetic testing showed that he was a carrier of the mutation in the SCNN1A gene. The second sibling developed salt wasting with Na^+^ 131 mEq/L, K^+^ 7.1 mEq/L, and HCO_3_^−^ 13 mEq/L at 4 days of life. Physical exam showed a dusky appearing newborn with poor tone. She was started on Kayexalate (3 grams every 8 hours) in addition to NaCl and NaHCO_3_ supplements at a total dose of 60 mEq/kg/day of sodium. Genetic testing revealed the same SCNN1A mutation as her sister described above, confirming the diagnosis of systemic PHA type I. Her course was uncomplicated and she was discharged from the NICU at 10 days of life. Sodium doses were titrated based on serum levels with eventual decrease to 26 mEq/kg/day of sodium by 21 days of life.

## 3. Review of Literature

### 3.1. Clinical Presentation

Systemic PHA type I typically presents in the neonatal period with severe salt wasting crisis with hyponatremia, hyperkalemia, acidosis, and dehydration due to sodium loss through the kidney, colon, sweat, and salivary glands [4]. Patients may also present with lethargy and failure to thrive. Hyponatremia and hyperkalemia are combined with elevated plasma renin activity and aldosterone levels reflecting renal resistance to aldosterone. Recurrent salt wasting crises and severe hyperkalemia can lead to life-threatening cardiac arrhythmias and cardiac arrest. Patients may present with symptoms similar to cystic fibrosis such as elevated sweat chloride secretion, failure to thrive, and recurrent pulmonary infections [24]. In systemic PHA type I defects in the ENaC channel lead to decreased sodium-dependent liquid absorption, which leads to excess liquid in the airway lumen causing narrowing of the airway and predisposing patients to wheezing and airway infections [24]. Systemic PHA type I has also been reported to present with pustular miliaria rubra, which is characterized by itchy eruptions affecting mainly the trunk and limbs appearing within the first few months of life [25]. This is thought to be due to sweat gland duct occlusion and inflammation of eccrine glands as a result of sodium accumulation, as ENac is expressed strongly in eccrine glands as well as multiple epidermal layers [25, 26].

### 3.2. Differential Diagnosis

Initial presentation of systemic PHA type I may be confused with salt wasting congenital adrenal hyperplasia (CAH), renal PHA type I, secondary PHA, or renal tubular acidosis (RTA) type IV. Salt wasting CAH due to 21-hydroxylase deficiency leads to cortisol and aldosterone deficiency. Aldosterone deficiency leads to the same presenting symptoms as the aldosterone resistance that is characteristic of systemic PHA type I. Females with salt wasting CAH are born with virilized genitalia due to elevated androgen levels, but males with salt wasting CAH have normal genitalia. Patients with systemic PHA type I typically have normal genitalia, making males presenting with salt wasting particularly difficult to differentiate between CAH and systemic PHA type I based on clinical presentation alone. Renal PHA type I may present with similar symptoms as systemic PHA type I, but it is less severe, limited to renal salt wasting, and symptoms improve with age [6]. A positive sweat or salivary sodium level can confirm the diagnosis of systemic PHA type I and can differentiate this from renal PHA type I. Secondary PHA is transient aldosterone resistance secondary to a urinary tract infection (UTI) [7] or urinary tract malformation [8, 9]. Nandagopal et al. report 4 infants with UTIs who developed severe hyponatremia and were diagnosed with transient PHA thought to be secondary to UTI [7]. Treatment with intravenous saline and antibiotics corrected the hyponatremia in these patients within 24–28 hours. Urinary tract malformations also led to secondary PHA in 6 infants aged 9 days to 7 months with bilateral ureterohydronephrosis and vesicoureteral reflux, who developed renal aldosterone resistance with hyponatremia, hyperkalemia, and metabolic acidosis [8]. These symptoms improved after treatment of the underlying condition [8]. RTA type IV is characterized by low levels of ammonia in the urine and typically presents with mild hyponatremia, hyperkalemia, and metabolic acidosis with aldosterone deficiency or defective signaling [10].  Table 1 summarizes the biochemical characteristics of these different conditions.

### 3.3. Genetic Diagnosis

Systemic PHA type I is caused by an inactivating mutation of the gene encoding ENaC. The channel is composed of three related subunits (alpha, beta, and gamma) encoded by three genes [4]. The alpha subunit gene SCNN1A is located on chromosome 12, whereas the beta subunit gene SCNN1B and the gamma subunit gene SCNN1C are located on chromosome 16. Worldwide more than 40 different mutations have been described in the coding regions of ENaC subunit genes [2, 13, 27, 28]. The majority of mutations appear in the alpha subunit gene SCNN1A, most frequently in exon 8 [14]. Mutations are nonsense, single base deletions or insertions, or splice-site mutations, leading to abnormal length mRNA and proteins [14]. Few novel missense mutations have also been reported [11, 13, 15]. Only a few cases of beta and gamma subunit gene mutations have been reported [29]. Phenotype and genotype correlation has been noted with a more severe phenotype in nonsense, frameshift, and abnormal splicing mutations compared to patients with missense mutations [2, 14, 15].

### 3.4. Treatment

Patients with systemic PHA type I often present with severe salt wasting crisis, so prompt management is needed to prevent severe illness. In the acute phase patients require intravenous fluids for dehydration as well as intravenous sodium chloride to treat hyponatremia. Sodium requirement is generally very high, up to 15–20 grams/day and even 45 grams/day in one report [30] compared to the much lower requirement of 1–3 grams/day in salt wasting CAH or renal PHA type I [31]. Intravenous sodium bicarbonate is used to correct metabolic acidosis and to improve hyperkalemia [31]. Severe hyperkalemia may require treatment with sodium polystyrene sulfonate resin therapy (Kayexalate), calcium carbonate, intravenous insulin and glucose, or transient peritoneal dialysis in severe cases [31]. In the newborn period, it is recommended that polystyrene therapies should be administered orally, as rectal administration has the risk of causing colonic necrosis and perforation [31]. Long-term treatment consists of oral sodium chloride supplementation for hyponatremia, oral sodium bicarbonate for metabolic acidosis as well as additional sodium supplementation, and oral Kayexalate to prevent hyperkalemia. Infants that have difficulty tolerating these treatments may require gastrostomy tube placement [31]. Although fludrocortisone is used for treatment of CAH, it is not beneficial in systemic PHA type I due to the dysfunction of the ENaC channel causing resistance to aldosterone and fludrocortisone.

### 3.5. Follow-Up Data

Limited data is available related to the long-term disease course of patients with systemic PHA type I.  Table 2 summarizes patients reported with systemic PHA type I in the literature and the follow-up data available [11–23].

The majority of reported cases of systemic PHA type I presented in the first two weeks of life, with age at presentation ranging from 1 to 17 days [11–23]. Duration of documented follow-up ranges from 5 months to 22.1 years [11–23]. Most patients continue to require significant doses of sodium supplementation although at least two reported cases were able to discontinue sodium supplementation [13, 18]. Several patients required gastrostomy tube placement to prevent salt wasting crises [14, 20]. The majority of patients with systemic PHA type I have short stature although several did attain average heights; however their weight varies widely. Respiratory symptoms were frequently reported but decreased significantly with age, and there is no clear relationship between existence of respiratory symptoms and specific mutations [11–23].

Salt wasting episodes also tended to decrease in frequency and severity with increasing age, although the clinical course varied significantly among patients. Edelheit et al. reported a patient with a homozygous mutation in the beta subunit who required gastrostomy tube placement at 14 months of age for prevention of life-threatening salt wasting crises [14]. At age of 4 years, he was receiving 15 grams NaCl/day by gastrostomy. Potential mechanisms for this improvement in sodium homeostasis with age include partial activity of the mutated ENaC subunits, upregulation of the thiazide sensitive NaCl cotransporter, decreased reliance on aldosterone for sodium regulation with age, increased dietary salt intake, maturation of kidneys, and compensatory increase in proximal sodium reabsorption [13, 17]. However, the majority of patients continue to require sodium supplementation lifelong [2].

In addition, Hanukoglu et al. described the long-term follow-up of 4 patients, 3 with different homozygous mutations in the alpha and beta subunits and 1 with compound heterozygous mutation in the alpha subunit [15]. Though all patients demonstrated normalization of the urinary sodium and potassium with age, the patient with the compound heterozygous mutation had the least severe clinical course and required the lowest dose of sodium supplementation. The patients with homozygous mutations, on the other hand, experienced multiple salt wasting episodes and required repeated hospitalizations for respiratory symptoms, though the frequency decreased with age [15]. This suggests that the clinical severity of disease may be closely related to the type and location of the genetic mutation [2, 14, 15].

Schaedel et al. previously reported a patient with the same mutation as our patient described in this report [11]. This patient was diagnosed at 11 days of age with severe hyponatremia and hypokalemia. He required long-term treatment with sodium supplements and an ion-binding resin, though doses were not provided. He only had 2 reported episodes of bronchopneumonia by age of 9 years, which is less frequent than our reported patient and compared to other patients in their report [11].

## 4. Conclusion

In summary, systemic PHA type I is characterized by salt wasting crises in infants and neonates due to inactivating mutations in ENaC subunits. It most commonly presents with failure to thrive and dehydration, and diagnosis is made by laboratory evaluation and genetic testing. Symptoms may persist into adulthood but often with decreased severity. With proper treatment and follow-up of systemic PHA type I, patients are expected to have normal physical and neuromotor development.

## Figures and Tables

**Table 1:** Biochemical characteristics of the conditions included in the differential diagnosis for systemic pseudohypoaldosteronism type I.

Condition	Serum Na^+^	Serum K^+^	Serum aldosterone	Plasma renin	Serum cortisol	Serum 17OH progesterone	Urine Na^+^
Systemic PHA type I	Low	High	High	High	Normal	Normal	High
Salt wasting congenital adrenal hyperplasia	Low	High	Low	High	Low	High	High
Renal PHA type I [6]	Low	High	High	High	Normal	Normal	High
Secondary PHA [7–9]	Low	High	High	High	Normal	Normal	High
Renal tubular acidosis type IV [10]	Low	High	Low	Low	Normal	Normal	Normal

**Table 2:** Compilation of clinical follow-up data of pseudohypoaldosteronism type I patients reported in the literature.

Author	Age at follow-up (years)	Mutation	Sodium requirement	Kayexalate dose	Height	Weight	Comments
Nur et al.	5.4	*SCNN1A 1685 C>T* Missense	23 mEq/kg/day	2.4 g/kg/day	−1.5 SDS	−3.1 SDS	Frequent respiratory infections from age of 2 to 3 years, improved with increasing age.

Schaedel et al. [11]							
*Patient 4*	9	*SCNN1A 1685C>T*; SCNN1A 1477 T>C; SCNN1A 1449delC Missense, compound heterozygous	NA	NA	NA	NA	Frequent salt wasting episodes; constipation and gastric ulcer; infrequent respiratory symptoms.
*Patient 1*	2	SCNN1A 1449delC Frameshift, homozygous	NA	NA	NA	NA	Respiratory infections starting at 8 months.
*Patient 3*	8	SCNN1A 729delA; SCNN1A 1449delC Frameshift, compound heterozygous	160 mEq/day [12]	10 g/day [12]	Short stature. [12]	NA	Requires antibiotics (often IV) for respiratory symptoms q4–8 weeks; recurrent pseudomonas pneumonias. [12]

Dirlewanger et al. [13]	0.5	SCNN1A c.727T>C Missense, homozygous	0	0	NA	NA	Born prematurely; transient PHA; able to discontinue salt supplementation at 6 months. Sibling born at term with identical mutation but no symptoms.

Edelheit et al. [14]							
*Patient 44*	8.3	SCNN1A 1078G>T; SCNN1A 1404delC	8 g/day	0	NA	NA	No salt wasting episodes or respiratory tract infections after discharge at 4.5 months; Kayexalate discontinued at 15 months.
*Patient TR2*	0.4	SCNN1A 1455delC Frameshift, homozygous	3 g/day	12 g/day	NA	NA	Developing normally.

Hanukoglu et al. [15]							
*Patient A*	11	SCNN1A 327G>C; SCNN1A His450fs Compound heterozygous	8 g/day	NA	0.4 SDS	0.9 SDS	No salt wasting after age of 9 years; no respiratory symptoms.
*Patient B*	20	SCNN1A Arg508 Stop, homozygous	15–20 g/day	NA	−2.1 SDS	0.4 SDS	Decreased frequency and severity of salt wasting episodes and respiratory symptoms with age.
*Patient C*	22.1	No mutation found	<10 g/day	NA	−1.8 SDS	−0.1 SDS	No severe salt wasting or respiratory infection requiring hospitalization in the past 7 years despite poor compliance.

Ekinci et al. [16]	3.25	SCNN1A c.684+2 T>A Splice-site, homozygous	21.8 mEq/kg/day	3.4 g/kg/day	0 SDS	+2 SDS	No respiratory problems or hospitalizations since neonatal period.

Dogan et al. [17]	3.5	SCNN1B c.1266-1G>C Splice-site, homozygous	26 mEq/kg/day	1.5 g/kg/day	−3.1 SDS	−1.3 SDS	Last hospitalization (for salt wasting and bronchopneumonia) at 11 months.

Adachi et al. [18]	20	SCNN1G 1627delG, SCNN1G 1570-1G>A Compound heterozygous	0	0	−2.2 SDS	NA	Discontinued salt supplementation at age of 11 years; one further hospitalization and IV outpatient therapy ×3 since that time.

Schweiger et al. [19]	1	SCNN1A Nt505delAC Frameshift, homozygous	11 mEq/kg/day NaCl 12 mEq/kg/day Na Citrate	NA	5th percentile	1st percentile	Mild oxygen requirement with chronic cough.

Belot et al. [20]							
*Patient 1*	3	SCNN1B c.637C>T/p Gln213stop, homozygous	50 mEq/kg/day	NA	50th percentile	50th percentile	Fed by gastrostomy; frequent severe dehydration attacks.
*Patient 2*	5	SCNN1A c.1621C>T/p Arg508stop, homozygous	30 mEq/kg/day	NA	50th percentile	50th percentile	Repeated severe dehydration episodes and bronchitis attacks.
*Patient 3*	0.67	SCNN1G c.1318C>T/p Arg440stop, homozygous	27 mEq/kg/day	NA	50th percentile	20th percentile	Severe hyperkalemia at 5 months after gastroenteritis.

Thomas et al. [21]	7	SCNN1B Deletion in promoter region, homozygous	NA	NA	NA	NA	Significant respiratory symptoms with frequent hospitalizations when young; by age 7 years only symptom was exercise-induced cough.

Saxena et al. [22]							
*Patient 11*	15	SCNN1A 1621C>T Homozygous	NA	NA	NA	NA	Still requiring high amounts of sodium supplementation.
*Patient 14*	6.5	SCNN1A 1669 +1 G>A Splice-site, homozygous	16 g/day [14]	NA	10–25th percentile	10–25th percentile	Repeated salt wasting episodes with decreasing frequency with age; died of cardiac arrest at age 6.5 years.
*Patient 16*	4	SCNN1A 1439insT	150–180 mEq/day	15 g/day	NA	NA	Stable.

Welzel et al. [23]							
*Patient 1*	17	SCNN1A c.587_588insC Frameshift, premature stop, homozygous	11.5 mEq/kg/day	NA	Normal height	Obese	Recurrent salt wasting crises; cystic fibrosis-like phenotype with bronchiectasis.
*Patient 2*	11.5	SCNN1A c.1342_1343insTACA Frameshift, premature stop, homozygous	NA	NA	Normal height	Normal weight	Salt wasting crises occurring once a year; no pulmonary symptoms.
*Patient 3*	1.75	SCNN1A c.742delG Premature stop, homozygous	19 mEq/kg/day	0.4 g/kg/day	−1.87 SDS	−2.15 SDS	Recurrent mild prolonged respiratory tract infections; eczema-like skin lesions.
*Patient 4*	3.8	SCNN1A c.587_588insC Frameshift, premature stop, homozygous	20–28 mEq/kg/day	NA	−2.17 SDS	NA	Gastrostomy tube placed for failure to thrive; 2 pulmonary infections.
*Patient 5*	1.7	SCNN1A c.1474C>T Missense, premature stop, homozygous	34 mEq/kg/day	NA	−0.7 SDS	−1.2 SDS	Recurrent salt wasting crises; cardiac arrest at age of 11 months during a crisis; pulmonary infections; mild rashes.
*Patient 6*	0.9	SCNN1A c.189C>A Premature stop, homozygous	17 mEq/kg/day	NA	NA	NA	Neuromotor developmental delay; recurrent pulmonary infections; atopic-dermatitis-like rash; died of electrolyte imbalance during sepsis at age 11 months.
*Patient 7*	0.7	SCNN1A c.1361-2A>G Splice-site, homozygous	10 mEq/kg/day	NA	NA	NA	Hospitalized for hyponatremic convulsion; 4 episodes of severe bronchiolitis.

NA: not available.
